# Outer membrane vesicles as molecular biomarkers for Gram-negative sepsis: Taking advantage of nature’s perfect packages

**DOI:** 10.1016/j.jbc.2022.102483

**Published:** 2022-09-13

**Authors:** Lea Vacca Michel, Thomas Gaborski

**Affiliations:** 1 School of Chemistry and Materials Science, Rochester Institute of Technology, Rochester, New York, USA; 2 Department of Biomedical Engineering, Rochester Institute of Technology, Rochester, New York, USA

**Keywords:** extracellular vesicles, sepsis, outer membrane, antibiotics, biomarker, Gram-negative bacteria, outer membrane vesicles, EV, extracellular vesicle, IEC, ion exchange chromatography, LPS, lipopolysaccharide, OMV, outer membrane vesicle, SEC, size-exclusion chromatography, UC, ultracentrifugation

## Abstract

Sepsis is an often life-threatening response to infection, occurring when host proinflammatory immune responses become abnormally elevated and dysregulated. To diagnose sepsis, the patient must have a confirmed or predicted infection, as well as other symptoms associated with the pathophysiology of sepsis. However, a recent study found that a specific causal organism could not be determined in the majority (70.1%) of sepsis cases, likely due to aggressive antibiotics or localized infections. The timing of a patient’s sepsis diagnosis is often predictive of their clinical outcome, underlining the need for a more definitive molecular diagnostic test. Here, we outline the advantages and challenges to using bacterial outer membrane vesicles (OMVs), nanoscale spherical buds derived from the outer membrane of Gram-negative bacteria, as a diagnostic biomarker for Gram-negative sepsis. Advantages include OMV abundance, their robustness in the presence of antibiotics, and their unique features derived from their parent cell that could allow for differentiation between bacterial species. Challenges include the rigorous purification methods required to isolate OMVs from complex biofluids and the additional need to separate OMVs from similarly sized extracellular vesicles, which can share physical properties with OMVs.

According to recent retrospective studies, sepsis is not only the most expensive condition treated in US hospitals (1, 2) but also a leading cause of death (3, 4, 5, 6, 7). There are close to one million admitted sepsis cases in the US each year, with numbers rising year over year (2, 7). Sepsis occurs when host proinflammatory immune responses become abnormally elevated due to a dysregulated or aberrant host response to infection (8). In severe cases, sepsis can result in organ failure and death (4).

Diagnostic methods for sepsis can vary between hospitals, but often involve scoring systems (*e.g*., APACHE II and SOFA) that grade the severity of illness in patients (9). Many of the altered physiological parameters measured by these scoring systems are not necessarily specific to sepsis, which makes it difficult to diagnose sepsis in early stages. The timing of a patient’s sepsis diagnosis is often predictive of their clinical outcome, thus underlining the need for a more definitive molecular diagnostic test (2, 10, 11). In the last decade, with the increase in our understanding of the pathophysiological mechanisms behind sepsis, there has also been an increase in the identification of potential biomarkers for diagnosis (12, 13, 14, 15). However, there is still no gold standard diagnostic biomarker for sepsis, and only a handful of biomarkers are commonly used in hospitals today (12, 13, 14, 16, 17). This apparent contradiction between biomarker discovery and implementation is likely due to the incredible diversity and complexity of the causal organisms of sepsis (bacterial, fungal, or viral), the cascade of immunological responses to infection, and the pathophysiological mechanisms of disease for individual sepsis patients.

A recent (2018) retrospective observational study looked at 2,566,689 sepsis cases from the Premier Healthcare Database, which included data from ∼20% of US inpatient discharges among private and academic hospitals (2). The study found that a specific causal organism could not be determined in the majority (70.1%) of sepsis cases, likely due to aggressive antibiotics or localized infections (2). Among the causal organisms identified, the primary included *Escherichia coli*, other Gram-negative bacteria, and *Streptococcus* (2). In a separate smaller study of neonatal sepsis patient samples (*n =* 70), only 41% of blood cultures were positive for bacteria, but that number rose to 91% when the blood was tested using a more sensitive 16S rDNA quantitative PCR assay, suggesting that even in neonates, blood cultures, especially those procured after antibiotic treatment is initiated, are not a reliable determinant of bacterial infections (18).

The most recent Surviving Sepsis Campaign article, written as part of an international collaboration to provide evidence-based treatments and best practices to reduce mortality related to sepsis, recommends the initiation of antimicrobial treatment within 1 or 3 h of disease recognition for patients with and without possible septic shock, respectively (11). However, the article also emphasized the importance of identifying the causal organism(s) of infection, especially before considering longer term antimicrobial usage. For example, the team recommended, “*continuously re-evaluating and searching for alternative diagnoses and discontinuing empiric antimicrobials if an alternative cause of illness is demonstrated or strongly suspected* (11).” This recommendation, combined with the common occurrence of falsely negative blood cultures, underlines the need for a fast and reliable method for detecting and identifying bacteria in sepsis patient biofluid.

Later, we introduce the concept of extracellular vesicles (EVs) as uniquely qualified to serve as molecular biomarkers for the diagnosis of bacterial sepsis due to their conserved, native-like content, their association with the host inflammatory response, and their robustness (and potential enhancement) in the presence of antibiotics. Due to their size, EVs are known to widely circulate in the body and in some cases readily cross tissue barriers, enabling potential diagnosis from easily accessed biofluids such as blood or urine.

## EVs as molecular biomarkers for sepsis

**Figure 1:**
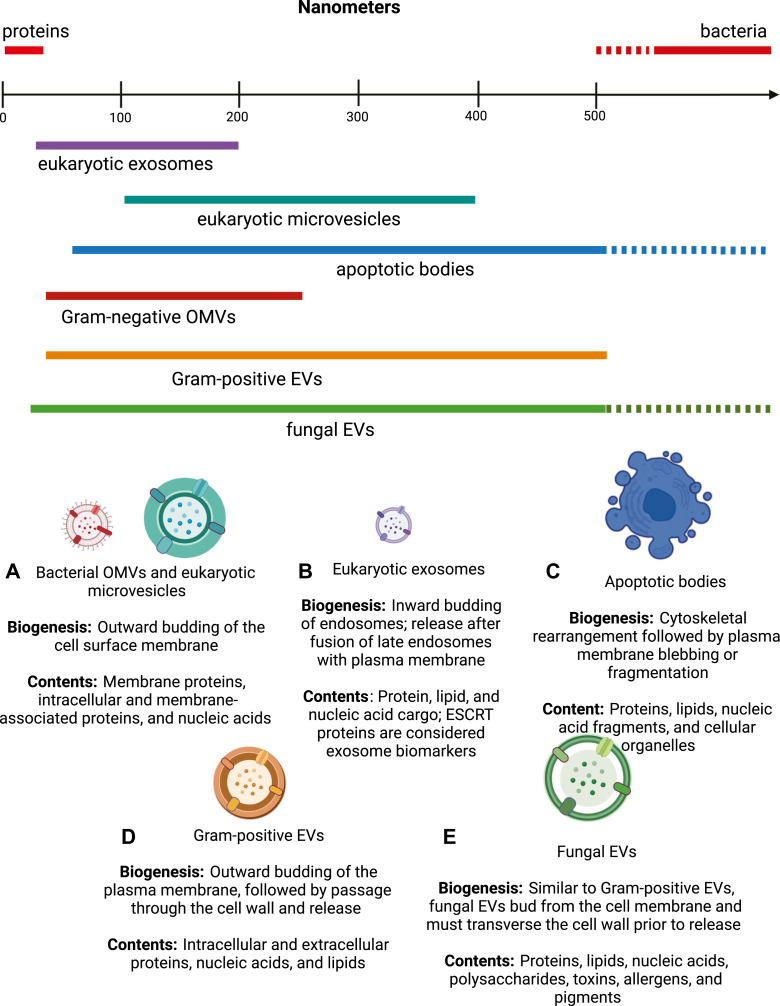
**Types of extracellular vesicles (EVs).***A*, bacterial OMVs and eukaryotic microvesicles are formed when the surface membrane is curved and then pinched off, releasing the spherical vesicle. These EVs, therefore, have similar surface properties to their parent cells. *B*, eukaryotic exosomes are intraluminal vesicles that are released upon fusion to the plasma membrane, allowing for the transport of cell-specific cargo to neighboring or distant cells. *C*, apoptotic bodies are a byproduct of programmed cell death, ranging from 50 to 5000 nm in diameter and containing nuclear fragments, cellular molecules, and organelles. *D*, Gram-positive EVs are produced from the pinching-off of the inner membrane, carrying a diverse array of cellular cargo to the extracellular space but not before transversing through the thick cell wall. *E*, fungus can release EVs in the form of smaller exosomes and larger microvesicles (up to 2000 nm in diameter). Like their counterparts, fungal EVs contain a variety of cellular contents, useful for intercellular communication.

EVs are nanoscale, lipid-bound species released from both prokaryotic and eukaryotic cells, which contain a multitude of cellular components, including intracellular soluble and membrane-associated proteins and nucleic acids, all originating from the parent cell from which they derive (Fig. 1). There are many proposed functions of EVs, including those related to intercellular communication and quorum sensing, pathogenesis, disease state regulation, and cellular survival (19, 20).

EVs originating from eukaryotic and particularly human cells have been long studied with significant growing interest in recent years, but the term EV has been loosely used in the literature. In 2014, the International Society of Extracellular Vesicles (ISEV) established guidelines titled Minimal Information for Studies of Extracellular Vesicles to standardize protocols, nomenclature, and reporting, updating these guidelines again in 2018 (21, 22). The major classes of EVs originating from eukaryotic cells are defined by their biogenesis. Intraluminal vesicles originating in multivesicular bodies that are ultimately released upon fusion of these bodies with the plasma membrane are called exosomes (Fig. 1*B*). Microvesicles (Fig. 1*A*) are EVs that are regularly shed directly from the cell membrane upon outward budding. Due to their biogenesis, the lipid and membrane composition of exosomes and microvesicles differ as well as their luminal contents. Because a microvesicle results from the budding of the plasma membrane, its lipid composition, membrane-bound proteins, and surface markers closely mimic the surface of the parent cell. The third major class of EVs, apoptotic bodies (Fig. 1*C*), are released in the final stages of apoptosis through blebbing of the plasma membrane and have similar membrane composition to microvesicles. While exosomes are often the smallest class of EVs at just 20 to 200 nm in size, they do overlap in size with microvesicles, commonly 100 to 400 nm, as well as larger apoptotic bodies (50–5000 nm) (22, 23).

### Bacterial EVs

Bacterial cells are known to release EVs, and their biogenesis is similar to EVs from human cells but with distinct differences, particularly between Gram-positive and Gram-negative cells (20). EVs originating from Gram-negative bacteria have been studied for decades and are most commonly referred to as outer membrane vesicles (OMVs). OMVs (Fig. 1*A*) are similar to eukaryotic microvesicles in that they result from the pinching off of the outer membrane. Gram-positive cells with a thick cell wall were not initially thought to be capable of releasing EVs, but an increasing number of studies have demonstrated their existence (24, 25, 26, 27, 28). Gram-positive EVs (Fig. 1*D*), and similarly fungal EVs (Fig. 1*E*), bud from the inner membrane and must travel through the cell wall (also known as the peptidoglycan layer) prior to their release. Some studies suggest that a weakening of the peptidoglycan layer may enable and promote EV release (24, 25, 26, 27, 28).

OMVs are generally similar in size to eukaryotic exosomes and smaller microvesicles, typically between 20 to 250 nm in diameter. They function to secrete cellular components as a way of promoting pathogenesis, surviving stress conditions, or regulating microbial interactions within bacterial communities (29). OMVs contain surface proteins, intracellular proteins, nucleic acids, pieces of peptidoglycan, and other cellular materials, and they have a similar bilayer outer membrane as their parent cell, with lipopolysaccharide (LPS) in its outer leaflet and an inner leaflet composed of phospholipids (29, 30).

### Advantages to using OMVs as biomarkers for Gram-negative sepsis

**Figure 2:**
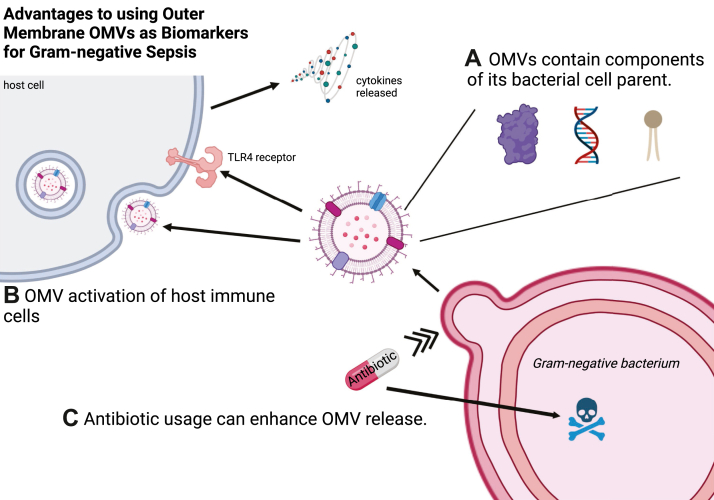
**Advantages to using OMVs as biomarkers for Gram-negative sepsis.***A*, OMVs contain biomolecules, including outer membrane and periplasmic proteins, nucleic acids, LPS, and other lipids, that are similar to its bacterial cell parent, allowing for the possible identification of the bacterial source of infection. *B*, OMVs can activate host immune cells, triggering the release of proinflammatory and anti-inflammatory cytokines and are capable of self-entry deep into host tissues, resulting in longer term, chronic inflammatory pathologies. *C*, the broad spectrum use of antibiotics to treat sepsis can lead to negative bacteria cultures. However, OMVs, which are continually released by bacteria and often enhanced in the presence of antibiotics, may offer an alternative method for identifying the causal organism of infection. OMV, outer membrane vesicle.

Here, we describe three major advantages to using OMVs as biomarkers for Gram-negative sepsis: their ability to induce host inflammation and their probable role in bacterial sepsis (Fig. 2*B*), their parent-derived antigenic content (Fig. 2*A*) (although as discussed later, specific antigenic content can change with environmental conditions), and their robustness in the presence of antibiotics (Fig. 2*C*). While this review focuses on Gram-negative OMVs, we propose that EVs from Gram-positive bacteria may be similarly effective biomarkers for Gram-positive sepsis, allowing for possible differentiation between bacterial species and identification of the causal organism(s) for all cases of bacterial sepsis.

Scientists have demonstrated that OMVs are capable of initiating the inflammatory response seen in the transition of an infection to sepsis, play a complex role in endothelial activation, and can induce cardiac injury, a sepsis complication that can worsen patient outcomes (31, 32, 33, 34, 35). OMVs contain toxins, virulence factors, adhesins, and immunomodulatory compounds, contributing to bacteria–host interactions and capable of inducing the host inflammatory response (36). Bacterial OMVs interact with host cells through several different mechanisms, such as activating host immune cells *via* TLRs (*e.g*., TLR4), triggering the release of proinflammatory and anti-inflammatory cytokines, and delivering bacterial content into host cells (37, 38, 39, 40). The delivery of toxins inside OMVs has several advantages. First, OMVs are capable of self-entry deep into host tissues, engaging both the innate and adaptive immune systems and resulting in longer term, chronic responses and inflammatory pathologies (36, 41, 42, 43, 44). Additionally, membrane-enclosed OMVs offer at least partial protection from degradation *via* host proteases (45, 46, 47), and OMVs can simultaneously deliver a variety of bacterial molecules, including LPS and other inflammation-inducing lipoproteins (48). OMVs can also trigger mitochondrial apoptosis and the inflammasome pathway in macrophages and dendritic cells (38, 39, 49, 50). And finally, OMVs have been shown, in mice, to induce disseminated intravascular coagulation, a severe complication of sepsis that significantly increases the probability of mortality in septic patients (51, 52).

LPS, also known as bacterial endotoxin, is the major lipid component in the outer leaflet of Gram-negative OMVs and is thought to be a major player in the induction of Gram-negative sepsis-related inflammation (53). While a clinical trial using mAbs to LPS alone did not significantly protect against the lethality of *E. coli* (*E. coli*) sepsis, an antisera therapy containing antibodies to several outer membrane proteins, later identified as OmpA, Lpp (Braun’s lipoprotein), and peptidoglycan-associated lipoprotein (Pal), did yield significantly protective results in sepsis patients compared to placebo (54, 55, 56, 57, 58, 59, 60, 61, 62). *E. coli* OMVs contain LPS, OmpA, Lpp, and Pal, all of which have been shown to be released from *E. coli* as a complex in the presence of human sera and antibiotics, as well as in several animal models of sepsis (61, 62, 63, 64, 65, 66, 67). Additionally, Pal and Lpp have been shown to be inflammatory and to contribute to virulence on their own and in combination with LPS (64, 68, 69, 70, 71, 72, 73). These results and the studies described previously all point to OMVs and their contents as significant contributors to the pathophysiology of Gram-negative sepsis.

The diversity in functional roles of OMVs suggests that the inclusion of specific OMV cargo would be a highly controlled and orchestrated event. However, the mechanism behind the incorporation of specific molecules into OMVs has yet to be elucidated. OMVs typically contain a variety of outer membrane and periplasmic components, and several studies have demonstrated both the enrichment and exclusion of specific protein cargo (compared to concentrations in whole bacteria), suggesting there may be a cargo selection process or that the mechanism of OMV formation results in the enrichment and exclusion of certain molecules (74, 75). For example, the oral pathogen *Porphyromonas gingivalis* is thought to selectively sort outer membrane proteins into OMVs, enriching them with virulence factors, by accumulating the molecules into microdomains of the outer membrane that are primed for vesiculation (76). Similar microdomains have been proposed by others, formed by the accumulation of misfolded proteins or by their lack of linkages (*via* Lpp or Pal, for example) between the outer membrane and the peptidoglycan layer (29, 75, 77).

While the mechanism of such cargo selection is still unknown, it is commonly accepted that OMVs contain components of their parent bacterium, which vary depending on growth conditions, including environmental stressors, and growth stage, which can affect the size and composition of the vesicles, as well as the expression and availability of proteins (75, 78, 79, 80, 81, 82, 83, 84, 85). Cargo that are conserved among a bacterium’s OMVs, independent of growth conditions and stage, would serve as ideal biomarkers, as well as cargo specific to a given bacterium that would allow for rapid identification and differentiation between bacterial species. Therefore, in order to utilize OMVs as effective biomarkers for sepsis, cargo that is conserved among the bacterial OMVs of interest must first be identified. At a minimum, LPS should be able to serve as a detectable biomarker for Gram-negative bacteria due to its abundance in the outer leaflet of the OMV outer membrane. And although outside the scope of this review, in the case that few or no proteins are conserved across species-specific OMVs, the amplification and detection of nucleic acids may be an alternative method for identifying the parent bacterial source of infection (86), especially considering the unique protection afforded to nucleic acids contained within membrane-bound OMVs.

As described previously, one of the biggest challenges in diagnosing sepsis and identifying the causal organism is the common occurrence of falsely negative blood cultures, due in part to the presence of antibiotics (wherein the bacterial infection source is unable to grow due to bactericidal or bacteriostatic levels of antibiotics in the host) and the inherent challenges in culturing bacteria *in vitro* (87, 88, 89). Despite these challenges and the additional hurdle of bacterial cultures taking up to 24 h for results, lab culture testing remains the most common method for identifying the causal organism of infection (11, 90, 91). As an alternative, OMVs and their parent-like features could be used to identify the bacterium. Unlike their bacterial parent, OMVs can withstand the inundation of most antimicrobials. Since bacteria release OMVs as part of their stress response, many bacteria have been shown to enhance OMV production and release in the presence of antimicrobials, such as gentamicin (92), antimicrobial quinolone PQS (93, 94, 95), polymixin B (94), ciprofloxacin (96), mitomycin C (97), and other antibiotics, especially those known to target the outer membrane, peptidoglycan, or LPS (67, 98, 99). OMVs are also thought to be released by bacteria to act as decoys, absorbing antimicrobials and antibodies so that the bacteria itself can evade the host’s innate and adaptive immune responses (99, 100).

Taken together, these advantages suggest that OMVs, which are continually released by Gram-negative bacteria and enhanced in the presence of environmental stressors, would allow for more efficient identification of the bacterial source of infection, even in the presence of antibiotics. However, before OMVs can become a reliable biomarker for Gram-negative sepsis, several significant challenges must be addressed, as described in the following section.

### Challenges to using OMVs as biomarkers and possible strategies to overcome them

The short- and long-term prevalence of OMVs in biofluids postinfection is still relatively unknown and understudied. We can surmise that bacteria will continuously release OMVs during the infection process, but post-infection, while the dysregulated inflammatory response wreaks havoc in the patient, how long will OMVs remain in circulation before being filtered out by the body? And compared to the patient’s own EV population, which includes EVs released by organisms in the host micro/mycobiome, how many OMVs will circulate, and how quickly do those numbers change during disease progression? Further, can we count on a detectable level of OMVs to be released into the bloodstream, independent of bacterial source and/or level of infection? One thought is that even very low levels of OMVs may be detectable and allow for identification of the bacterial source, although more sensitive detection techniques may be required, such as PCR-based methods. While exogenous nucleic acids from the bacteria will be quickly degraded by host nucleases, DNA or RNA contained within OMVs may be protected from degradation indefinitely. Effective isolation and purification of EVs from complex biofluids remain a challenge, and solving this will enable a variety of diagnostic strategies that will improve our understanding of sepsis progression and treatment for patients.

To detect OMVs in a complex biofluid such as human plasma, one must consider the OMV titer during infection and the sensitivity and selectivity of the detection device. Low titers of OMVs and/or low sensitivity of the detection device (*e.g*., weak antibody binding to the antigenic OMV target) could result in a weak positive signal, and high titers of extraneous EVs or poor selectivity of the detection device (*e.g.*, nonselective antibody binding to non-OMV targets) could result in false-negative results. Therefore, an initial purification and/or concentration step of the OMVs could allow for improved sensitivity and selectivity.

Purification of human and bacterial EVs from complex biofluids such as serum and urine is technically demanding. In addition to whole cells and cellular fragments, these fluids contain many types of proteins, lipid complexes, extracellular RNA and DNA, as well as other biological nanoparticles with overlapping density and size (101, 102). More specifically, in addition to human extracellular vesicles, there will likely be a background level of vesicles released by bacteria from the host microbiome and fungi from the host mycobiome (103, 104, 105, 106). While the remainder of this review will focus on separation techniques required to isolate EVs, we acknowledge the additional challenge that comes with differentiating vesicles from the infection source and those derived from the host, including those produced by the host’s own micro/mycobiome.

**Figure 3:**
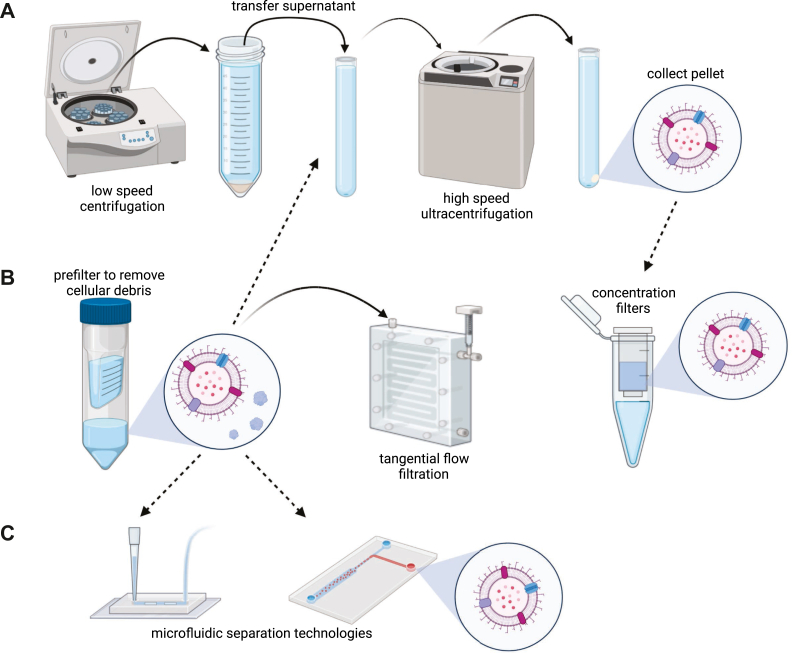
**Isolation and purification approaches for OMVs**. *A*, centrifugation is one of the most widely used techniques for isolating extracellular vesicles, including OMVs. The most common centrifugal approach is differential, which involves sequential centrifugation steps ending with ultracentrifugation. *B*, membrane filtration is also commonly used in the isolation of vesicles. A prefiltration step is used in several isolation approaches to rapidly remove large contaminants from small vesicles and proteins. Tangential flow filtration (TFF) is often used for high-volume isolation needs. *Low* molecular weight filters are sometimes used to concentrate vesicles after ultracentrifugation or chromatography. *C*, microfluidic devices incorporating novel isolation and separation technologies from acoustics to electrokinetics aim to improve recovery as well as separate vesicle subpopulations. OMV, outer membrane vesicle.

A variety of approaches have been employed to purify and separate EVs, ranging from centrifugation to novel microfluidic technologies (Fig. 3). Comprehensive reviews cataloging and comparing separation approaches have been published elsewhere (101, 102, 107, 108, 109). While most of these reviews have focused on human EVs, the same principles generally apply to OMVs. A summary of the most common techniques for isolating EVs and OMVs from septic patient biofluids and from each other are outlined later.

Two techniques became relatively common early on for the purification of EVs, ultracentrifugation (UC) and polymer precipitation. Precipitation with PEG was one of the first commercially available kits for EV isolation and gained relatively quick adoption because of its simplicity. Since the beginning of its use, however, several publications have shown that precipitation methods result in significant coprecipitation of contaminating proteins, lipoprotein complexes, and extracellular RNA, resulting in the discontinuation of this technique by many researchers (110, 111). On the other hand, UC remains a gold standard for the purification of EVs (Fig. 3*A*), although newer techniques are proving to offer higher purity and recovery (108, 112). Differential or sequential UC relies upon differing sedimentation rates of the biofluid constituents, typically recovering the supernatant and discarding the pellet until a final high speed centrifugation where EVs are ideally pelleted, while most proteins remain in the supernatant (113). Fundamentally, this technique cannot provide an absolute high purity separation as proteins, lipid complexes, and other non-EV species can aggregate and copellet. At the same time, high speed centrifugation can lead to fusion of the target EVs (114, 115, 116). Gradient UC can be performed after an initial purification to separate species that differ slightly in their density, even when similar in size, but some lipoproteins can still coisolate (117). Differences in biofluid viscosity, protein, and lipid concentration can dramatically alter UC results. For these reasons, EV researchers have sought solutions using a variety of other bioseparation approaches.

Several types of chromatography have been used to purify or isolate EVs (118, 119). Size-exclusion chromatography (SEC) has proven successful in separating smaller proteins from EVs, which are large enough to be excluded from the size-exclusion beads, passing more quickly into the collection fractions. Ion-exchange chromatography (IEC) has been used to target EVs that generally have a net negative charge. However, ion-exchange will also select for protein complexes with a similar charge. Vesicles that are not rich in negatively charged glycans, phosphoryl, and sulfo groups may not bind to the IEC matrix (120). Furthermore, the negative surface charge of most EVs can change as environmental conditions, such as pH, vary in the biofluids (121). Affinity chromatography can be used to select for specific surface markers of a particular EV subpopulation through the use of antibodies, aptamers, or other ligands (119). Unlike SEC, both IEC and affinity chromatography typically require elution buffers with significant changes in pH or ionic strength, which could affect the properties and functionality of the EVs. In all cases, chromatography solutions generally result in significant dilution, often requiring a final membrane concentration and buffer exchange step, which can lead to further loss.

A classic approach to purifying biomolecules from various biofluids is membrane filtration (Fig. 3*B*). Filtration can be applied in several manners depending on user requirements. Membranes have been used in EV purification pipelines in several ways (108), from prefiltration of cellular debris to concentration following chromatography to the separation of EVs from other small biomolecules using tangential flow filtration (122). Prefiltration often utilizes relatively large pores to allow EVs and proteins to pass while retaining cellular debris (123). The use of vacuum and syringe filtration membranes operating in normal flow, also known as dead-end filtration, is common, but prone to cake formation (accumulation of matter at the membrane surface) and significant loss in cell and protein-rich biofluids (124). Due to the substantial dilution that occurs during chromatography elution, EV-rich fractions are often combined and then concentrated with a low molecular weight cut-off membrane, where EVs are retained above the filter, while excess fluid passes through. EVs forced onto the membrane and into pores can be damaged and lost during this process (125). Finally, tangential flow filtration uses a sweeping process across the membrane to minimize cake formation and concentration polarization while passing smaller species, such as proteins, while retaining EVs (124, 126, 127). This approach is common in large-scale purification solutions but is gaining popularity in smaller formats due to higher purity and less loss compared to other filtration methods.

On the horizon are a number of promising technologies that rely on fluidic (asymmetric flow field-flow fractionation), electrokinetic, and acoustic focusing principles, often in combination with one or more traditional separation approaches (107). Many of these microfluidic technologies (Fig. 3*C*) have the potential to significantly improve purity with rapid processing times but are generally limited to relatively small sample volumes. Microfluidics can also enable the combination of size and affinity approaches in a single platform. While volume limitation may inhibit their widespread adoption for all EV isolation needs, improvements in purity and speed may be ideal for diagnostic purposes where sample volumes are relatively small.

In addition to the more generic challenges to isolating EVs, as described previously, isolating EVs from human serum offers its own unique challenges. Protein concentration in serum is typically very high (60–80 mg/ml) and can lead to almost immediate membrane fouling in normal or dead-end filtration modes used in vacuum or syringe filters. In contrast, tangential flow filtration can minimize this effect to maintain throughput (128). Serum also contains lipids such as high-density lipoprotein (7–13 nm) and low-density lipoprotein (18–23 nm) that are smaller than most EVs and can still be removed with size-based methods (101). High lipid concentrations, however, can foul some membranes and affect pellet formation during centrifugation. Larger lipids such as very low-density lipoprotein (30–80 nm) and chylomicrons (80–1200 nm) can overlap in size with EVs but have different densities. These nanoparticles can be separated from EVs using density gradient centrifugation (101, 129). Additionally, lipids and lipoproteins can be removed using affinity and, in some cases, charge-based separation techniques as discussed earlier. Urine has lower protein concentration, few lipid complexes, and overall, much lower viscosity than serum but also likely far fewer OMVs originating from sepsis and may not be a desirable or reliable source for these important biomarkers.

Separating EVs and bacterial OMVs from each other is yet another challenge, because they are similar in size. Physical separation approaches such as membrane filtration, SEC, and asymmetric flow field flow fractionation are not effective on their own. Separating vesicles based on surface antigens using affinity approaches is one possible strategy that could utilize chromatography, magnetic beads, or microfluidic capture on functionalized surfaces. Recently, some groups have successfully isolated human EVs by targeting specific phospholipids using Tim4 protein and annexin V (115, 130). Similarly, anti-LPS or LPS-binding effector TeoL could be used to isolate LPS-rich OMVs (131). Additionally, some are investigating whether proteins that sense and bind to highly curved phospholipid membranes and the peptides derived from them can be used to selectively capture EVs (132, 133). The net surface charge of OMVs likely differs from human EVs based on the variation in antigens, glycans, phospholipids, and the presence of LPS. The most successful strategy in isolating OMVs from human biofluids will likely use a combination of traditional bioseparation approaches, such as membrane filtration, in combination with vesicle-selective capture *via* affinity or charge interaction.

While a combination of isolation and detection approaches will likely be required, OMVs remain a highly attractive biomarker for Gram-negative sepsis diagnosis. The common occurrence of falsely negative bacterial cultures in sepsis patient biofluids continues to be a mystery, perplexing doctors and leaving patients with more questions than answers. Where bacteria fail (thankfully, due to stalwart antimicrobial treatments), OMVs may persist and allow for a more definitive diagnosis and a more targeted approach to treatment.

## Conflict of interest

The authors declare that they have no conflicts of interest with the contents of this article.
